# Dr. Harris, on the Yellow Fever

**Published:** 1803-01-01

**Authors:** John Harris

**Affiliations:** Kingston, Jamaica


To Dr. BRADLEY.

SIR,

THE just reputation which the Medical Journal has acquired for disseminating medical knowledge, will prove the best apology for my sending the following Observations, which I beg leave to submit to your approbation. The first part I deemed necessary to the introduction of the second; which is an humble attempt to be useful to my profession and my fellow creatures.

I am, &c.

JOHN HARRIS, M. D.

*Kingston, Jamaica, September* 13, 1802.

---

"Est quadam prodire tenus, si non datur ultra." HOR. EP.

"Dimidium facti qui cœpit habet." IBID.

The author who builds his hopes of acquiring popularity and fame on so flimsy a foundation as mere assertion, can little hope for stability in public confidence, when his assertions are opposed by facts. Every man who writes for publication, ought to ask himself this serious question: Am I to write for the good of mankind, or for my own gratification? If the good of mankind be the chief object of his endeavours, truth will guide his pen, and conscience approve his work. If self gratification be the ruling principle, vanity will lead him into error, which may not only mislead the present generation, but may extend its baneful influence to thousands yet unborn. The reader therefore may be assured, that whatever shall meet his eye in the following pages, has been dictated by candour, and will be supported by living evidences.

Long practice has made me fully sensible how little our best endeavours will often avail in surmounting the difficulties and dangers that present themselves almost at every step in the field of medicine. I am thoroughly aware of the numberless avenues that lead to death, and of the insufficiency of human means, and that too in many instances, when the arrogance of science has promised success! Yet there are means afforded by the great Creator to alleviate the sufferings of mankind; by the proper application of which we are enabled often to obviate the stroke of death. He who from attentive practice, experience, and faithful observation, exerts his abilities for the the good of his fellow-creatures, if they should not be crowned with success at all times, will always feel the reward of his honest endeavours. It would at this day be fortunate for mankind if medical men had always profited by the opportunities of observation, which a long and extensive practice affords; but it is to be lamented that there are practitioners, who, though they have been most favourably placed in this respect, are nevertheless so fettered by the trammels, and bigoted to the notions of former times, as not only to deceive themselves, but to spread the delusion among those whom ignorance, partiality, or fashion may have impressed with a false opinion of their abilities.

The malignant fever, which began its ravages here in 1793, has been erroneously denominated "The Yellow Fever." To distinguish a disease by any particular colour, implies that that colour prevails in every case; whereas, in this disease, the majority of cases met with exhibit no peculiar yellowness of the skin. The most characteristic mark of the malignant fever in question is a peculiar undescribable cast of countenance. All its other symptoms may be traced more or less in other fevers. The disease has been correctly described by Chisholm, Lemprierre, and others. The two first authors agree in the mode of treatment, but others differ very widely indeed; so as, in many instances, to recommend practices in direct opposition to each other! Perhaps, this may be accounted for from the consideration of climate; as it requires no great stretch of understanding to know that the same practice will not answer in all climates: a physician therefore is best qualified to judge of practice in that climate which has afforded him the most experience.

Although it might be expected that physicians, who have long sympathized with human sufferings, are most competent to account for their causes; yet it is much to be regretted, that all we have yet discovered serves but to shew, and oblige us to acknowledge, our ignorance; while we may hope that future discoveries in physiology will enable us to see more clearly into this subject.

Authors may differ in opinion in what they offer to the public; but there is a certain decency of expression towards the adverse party, and modesty respecting self, that ought to be observed in discussing a controverted point; which, while they preclude arrogance and prejudice, leave the reader at liberty to draw instruction from the exercise of his own judgment. A reader who has been in the habit of of judging for himself, will not be led away by the unqualified assertions and prejudice of an author, or his illiberal condemnation of a medicine; especially when he knows that medicine to have been, and is still, used with success under the strong recommendation of those who stand respectable in the medical world. But there may be some, particularly students and young practitioners in medicine, whose judgment cannot be supposed to secure them against the influence of an opinion, however erroneous, when it is asserted to be the result of long experience. Yet, though it is a matter of regret, that such opinion should be adopted without sufficient inquiry, he who does so is certainly less culpable, than he who from prejudice or dogmatic pride, not only continues a practice and opinion founded in error, but endeavours to impose them on the world.

When a malignant fever makes such dreadful ravages among mankind as to be justly called a new disease, not yielding to any former method of treatment, it becomes not only justifiable but absolutely indispensable to alter our mode of practice; and the physician who persists in an unsuccessful treatment, merely because he has adopted it at the commencement, and was not the first to use or recommend another more successful practice, will be deemed by all good men to be more actuated by pride and obstinacy, than by conscience or by benevolence to his fellow-creatures. To wait for a further improvement of the present imperfect state of our knowledge respecting this fever, without making known by degrees what we learn from experience, would be a delay not only dangerous to the lives of many, but highly derogatory to the duty we owe to society.

The high commendation given to the vigorous use of mercury in this fever, by such candid and able men as Chisholm and Lemprierre, ought to prepossess every one in favour of the utility and safety of the remedy, and prove a sufficient defence against any unqualified condemnation of it; yet it may justly be said, that although the practice has proved the best we at present know, it is much to be lamented that we have not been able to find a much better.

The love of truth alone obliges me to observe here, that the author of a late publication is neither correct nor charitable, when, writing against the mercurial treatment, he says, "I do aver, that altho' I have been called in to attend many under such circumstances, not one survived, and that they became more victims to the mercury than even to the fever."

fever." (Essay on Yellow Fever by D. GRANT, M. D. p. 51.)

The author, when he wrote this, could not have recollected the cases of Dr. C. M'Larty and I. M. Grant, Esq. both of whom he had occasion to see, and to prescribe for, under the circumstances he alludes to. The former is to be seen in his Thesis published at Edinburgh in 1797; the latter may probably appear at some future period.

If, on a comparison of the two modes of treatment, we find that many more recover by the use of mercury than by bleeding, or any other practice yet tried; surely every candid practitioner will allow, that the former is the most eligible and safest practice; and he will the more readily adopt this opinion, when he is informed that many here have observed such alarming symptoms often following the use of the lancet, as to induce them to condemn it as destructive.

Whatever opinion we may entertain of the *modus operandi* of medicines, whether satisfactory or otherwise, it should not influence our practice in a disease like this, in which we ought to be guided by our knowledge of the *juvantia et lædentia,* as derived from observation and experience. Ingenious theories look very well on paper; while they give celebrity to the author's acuteness, they will amuse the reader, and may exercise his imagination to reconcile them to practice. If this can be done, it will be no doubt both satisfactory and instructive. But if we cannot comprehend the principles, so as to form a rational theory on a successful practice, we ought neither to doubt of their correctness nor condemn the practice without a liberal trial.

The most learned physicians have not yet been able to penetrate farther than conjecture into the real cause of fevers in general: Nor shall I hazard an opinion on a subject, which has exercised the pens of so many celebrated writers. Though we may not be able to determine in what it consists, there is something peculiar in the constitution of people from a cold country, which renders them more obnoxious to fever in a warm climate, than either natives or those who have been assimilated to it by long residence. Accordingly we find, that the same exposure to the causes, predisponent and occasional, will produce fever in a stranger, while the native or old inhabitant remains in good health; and the symptoms will be tenfold more urgent in the one than in the other, supposing both are attacked. Hence it happens that long residents, and natives in general, are not at all liable to this fever: But when they are are attacked with the remittent of the country, the symptoms partake more or less of the malignancy of the prevailing epidemic; and require the aid of mercury to forward the cure with bark and the other medicines commonly in use.

I had a remarkable Case of this kind last year, in a young gentleman who had been all his life in a healthy cool situation in this island. He had been but a short time in town, when intemperance in living and exercise, together with exposure to other causes from the prevalence of the epidemic, threw him into a fever, which terminated fatally on the fifth day, in defiance of every thing that could be done for him. Indeed, I did not see him soon enough to try the effect of cold ablution. I thought this case much in favour of the opinion that the malignant fever is of a contagious nature.

The filth of Kingston has been always assigned as one cause of this epidemic. This, with the occasional prevalence of westerly winds, which bring with them marsh miasmata, may, no doubt, have considerable effect on the health of the inhabitants: But these are no new causes for a new disease. If this nuisance and marsh miasmata were the causes of the prevailing fever, why should a like pestilential fever rage on the Continent of America, particularly in cities where police is carried to the perfection of cleanliness? Whatever may be the cause, the dreadful scourge was inflicted nearly at the same period, both in America and in the West Indies.

There may be a peculiar quality in the blood of those strangers, who are the objects of this disease, which disposes the system to such general derangement, when attacked by fever, as no medicine can rectify: for we see, in many cases, when healthy action is restored, particularly in the liver, by the power of mercury, that the black stools become of the colour which the hepatic secretion always gives them; and that other dangerous symptoms vanish. Hence we may fairly conclude, that to attempt the assimilation of constitution to climate by a mercurial course before the attack, is a rational and safe practice, as shall be shewn hereafter: and, indeed, this becomes more indispensably the duty of the practitioners to recommend, and new comers to adopt, when they consider the general mortality attending this insidious disease, after it has been allowed to fix on the much endangered object.

In the true malignant fever now in question, there is not the least remission until it has entirely run through its first stage, stage, which is generally in thirty-six or forty-eight hours; when there is such an abatement of symptoms, as often to induce the patient to think himself well. Here the advocates for bleeding and bark, imagine they have nothing more to do than to administer the latter in large doses; and are led to pronounce a hasty opinion *of all danger being past.* But an early recurrence of the symptoms in an aggravated form, accompanied with extreme debility, bids defiance to bark, wine, blisters, and every other stimulant which can be given. If then this great debility and other symptoms of danger supervene in a rapid manner after one, two, or three bleedings, are we not to infer that, if the previous loss of blood had not occasioned, it had at least increased them? These (with occasional delirium) are the general effects of bleeding and bark, at the close of the unhappy scene! At other times the patient slips off, *without fever,* very unexpectedly. How can this be the remittent of the country, which has generally yielded to the bark?

From the torpor of the bowels at the beginning, and during the first stage of the disease, there may be some reason to suspect a deficiency of bile. The abstraction of its antiseptic quality from the bowels when in a healthy state, or its application to them when diseased, may occasion that flow of putrid stools which so frequently occur during the latter stage. That bile is a necessary stimulus to the bowels, communicating salutary effects to the system; that disease happens from deficiency in its secretions, from obstructions in the biliary ducts, or from increased action of the secreting vessels of the liver, whether arising from heat of weather or other causes; and that there may be congestion in the liver, both from accelerated and imperfect secretion of bile; I believe will not be denied by the most zealous opposers of mercury. That this medicine has been proved to possess the most stimulating and deobstruent qualities of any yet known, they cannot deny. Further, that many more are recovered by the mercurial treatment than by bleeding or any other practice, we not only learn from Dr. Chisholm's excellent work, from the practice in the fleet on this station, and the naval hospital at Port Royal; but from irrefragable proofs well known to all the practitioners, merchants, and others in the town of Kingston, from the commencement of that practice in 1794, to the present time.

Notwithstanding all the censure which has been bestowed on the prescribers of mercury in Kingston, the practice practice still obtains, to the total disuse of phlebotomy* in this disease. I shall think myself justifiable then in following the same course until a better be pointed out; on which event I shall freely adopt the better mode, and candidly acknowledge its superiority: For he who is open to conviction, and can so far conquer the pride of human nature as to relinquish a favorite opinion, although not founded in error, is as much farther advanced towards improvement, as he, who persists in obstinacy and error is receding from it.

These few desultory observations I have thought it my duty to make, in defence of a practice I have followed for eight years past. The subsequent part of this paper will be devoted to prophylaxis.

"Venienti occurrere morbo" is an old and very salutary maxim in medicine; and the more mortal the prevalence of a disease is, the more ought we to guard against it.

As mercury has been found in many cases to counteract the violence of malignant fever during its course, it was reasonable to expect that it would change that peculiarity of habit which rendered strangers obnoxious to the disease; and in a great measure prevent that combination of morbid action, which defied the power of medicine in so many instances. Accordingly, for four years past, I have prescribed it, with that view, for all who have applied, immediately or soon after their arrival, for advice; and the result has been such as to authorize the present attempt for the benefit of those who may in future visit the torrid zone. However, there are unfortunately many prejudices which I have found very difficult to conquer, in recommending this practice. These probably have originated as much from the contrariety of opinions delivered to the public, as from individual caprice. Some contend that they are in good health, and will not have it reduced by medicines; saying, that it will be soon enough to take physic when sickness renders it necessary. The minds of others have been prepossessed with the false notion of the bad effects of mercury on the human constitution; while others think that any preparation whatever is altogether nugatory; and that the best way to meet the effects of a relaxing climate is, *to live freely in order to support the strength of Nature.* Alas! that these are mistaken and devoted victims, every day too clearly evinces!

If

* See the annexed letter from Dr. Mac Larty.

If variolous inoculation has long since proved such a blessing to mankind, as to render that mortal disease, and even the plague itself, more tractable by the previous use of mercury; why may we not attempt by the same means, to control the mortal epidemic in question? If there had been but one instance of prevention or preservation from the malignant fever by means of mercury, that alone would be sufficient to recommend the practice; but when numbers of living examples can attest the fact, I trust that this communication will be received with that candour, which is due to good intention; and the practice adopted, both by medical gentlemen and emigrants, with that confidence which is warranted both by irrefragable proofs, and a certainty of its safety. The simplicity of the following directions may raise some scruples in weak minds; but it will be a recommendation to those of a contrary frame.

The plethoric and robust, being the subjects most liable to this disease, on their approach to the hot latitudes are to be bled in proportion to their strength. According to circumstances, of which medical men only are competent to judge, this may also be done immediately after their arrival. The bowels are then to be emptied by some cooling physic, and in some cases the stomach also, by means of an emetic. The same circumstances which regulated the quantity of blood to be drawn, will determine the dose of calomel to each individual, from two to five grains, every, or every other night, until the gums are affected. If it should run through the bowels, as is sometimes the case, a few drops of laudanum should be added to the dose. The best way of taking it, is in form of a powder, in some thick vehicle; pills often becoming so hard as to pass through the bowels unchanged. When the mouth becomes affected, a dose of cooling physic should be administered, after two or three days intermission of the mercury. In some constitutions not easily affected by mercury, it will be necessary to persevere with steadiness, until the system be thoroughly impregnated with the medicine; for thereon depends the safety of the patient. In every instance, temperance is to be strictly enjoined; and exposure to heat and cold to be sedulously avoided, until the patient becomes habituated to the climate.

If at any time, especially during the hot months, the new-comer should feel himself oppressed by heat, nothing will relieve him sooner than the tepid bath; that is, a bath as nearly of the temperature of the body as his feeling can determine. During this time, a cheerful confidence is is to be impressed on the mind, respecting the efficacy of the preparation towards lessening the danger of fever. This is highly necessary, in order to obviate the sedative effects of fear, which in many instances has proved a powerful agent towards fatal terminations, when there was no other alarming symptom.

The custom of early going to bed and early rising is conducive to health every where; but more especially in hot countries. If gentle exercise be added in the morning, it will forward the intention; particularly if the new-comer is obliged to live in town. Those who may have an opportunity of a country residence for some months should always embrace it; nevertheless the foregoing rules are to be observed, especially if they mean to return to town. In all situations, a costive habit of body is to be guarded against by the use of mild purgatives. This preparatory course, independant of malignant fever, will be found extremely salutary against complaints of the liver; so prevalent, and so often neglected, in this country.

It will be easily understood here, that bleeding, as a preparative, will have a very different effect from what it would have in a curative intention; for in the former, it prevents morbid action, and gives time for assimilation; whereas, in the latter, it induces debility, and confirms morbid associations, very dangerous to life.

If I have detained the reader too long, let my zeal for the service of my profession, and fellow creatures, plead my excuse. I shall only add that, although I cannot claim any praise for the execution of this attempt, my intention is entitled to some indulgence from the candid reader; and if this subject should be enlarged upon by some abler pen, so as to recommend the practice to general notice and adoption, I shall be sufficiently gratified in the hope that my attention may be fulfilled in the preservation of some lives from one of the most fatal diseases that ever afflicted mankind.

"Vive! vale! si quid novisti rectius istis;
"Candidus imperti; si non his utere mecum."

P. S. Trusting there are none, mentioned in this paper, who would not go much further than contributing their names in the service of truth and humanity, I need not apologize for the liberty of using them in support of a practice, which has been beneficial to themselves, and may prove so to many others.

The following were treated under my own inspection:

| | |
|---|---|
| 1. Mr. James Ore | 7. Mr. John Dwyer |
| 2. Mr. Robert Mac Glashan | 8. Mr. Dennis Dwyer |
| 3. Counsellor Kean | 9. Mr. James Keene |
| 4. Miss Young | 10. Mrs. Courtauld |
| 5. Mr. Thomas Geoghegan | 11. Mr. James Rawlins. |
| 6. Mr. David Clark | |

Of the above, the second died of angina maligna; the third, by a fall from his horse; and the eleventh, from inflammation of the bladder, some time after having gone through the fever safely. The rest are still alive and well.

A family of four persons, some months arrived, are now under preparation; one of whom has just recovered from the disease; and the rest are in perfect health.

---

*Extract of a Letter from* Dr. Charles Mac Larty, *to* Dr. Harris.

Dear Sir,

IN answer to your inquiries, I shall relate what has fallen under my observation with regard to the use of mercury as a preventive, and of the lancet as a remedy in the fever to which new-comers are subject.

I was induced by the hope of deriving benefit from mercury as a prophylactic, to give it to three passengers and several seamen on board the ship in which I returned to this island in the year 1797. As none of them had been before in a warm country, I was at pains to remove from their minds every dread they might entertain of the unhealthiness of the climate; stating, that it was in their power to obviate its influence, with respect to themselves, by prudent conduct in their mode of living, and in avoiding improper exposure; and, to inspire them with a confidence in mercury both as to its safety and utility, I myself began to take it at the time it was recommended to them. Example in this instance had a good effect, for some who at first expressed aversion to taking mercury on any account, afterwards requested that I would give it to them. Each person came to me every evening for his pill, which contained gr. jß. of calomel. Its use was commenced when about as far to the southward as Madeira, and continued so as to keep up a slight affection of the gums till we arrived off St. Domingo; when an accident which carried the ship into the harbour of Cape Nichola Mole on the 19th of October, rendered me unable to attend any longer to the prophylactic patients. From this till our arrival at

Kingston,

Kingston, on the 15th of November, three weeks of which the ship remained at the Mole, not a man was attacked with fever, though it was prevalent on board the ships of the navy, then lying there. This I ascribed more to the terror of the impress having kept our men on board, out of the way of contagion, and clear of the allurements of grog, and its baneful consequences.

As the ship's stay at Kingston after her arrival was short, and most of the people left her at the time, I know nothing of the subsequent history of those seamen who took mercury, two excepted. One had made a voyage to Britain, and told me that during the time he was in Jamaica he had no attack of fever; the other had been impressed into the navy, and, two years after, when I saw him, said he had ever since been in the West Indies and enjoyed good health.

Of the passengers, one has been always near, and in Kingston. Nine months after his arrival he was attacked with fever in a mild form; for the cure of which, mercury was administered. His recovery was speedy and perfect, and he has enjoyed good health ever since. From him I learn that another of the passengers resides at Port Antonio, quite well; and that he had fever, but not till three years after coming to Jamaica. The third passenger has always lived in an elevated and consequently healthy situation on a mountain, in the parish of St. Andrew, from whence he comes occasionally to Kingston for a few days; but has never complained.

To a young gentleman who was about to leave Britain in order to come to Jamaica, I gave directions by letter to begin a course of mercurial medicines immediately on his arriving in the warm latitudes. Though he most punctually attended to what was directed to be observed during the passage, and to every thing recommended after his arrival, he was nevertheless soon attacked with fever in a very aggravated form, and became one of its too numerous victims. This instance would appear to disprove the opinion which you have formed of the efficacy of mercury, as either preventing, or rendering milder, those attacks of fever to which new-comers are so generally subjected; but the instances before related certainly favor such an opinion. Supposing it acted as a prophylactic in only a few out of many trials, it would still be our duty to recommend the practice; and that more especially when it is considered, that by so doing we may relieve strangers from that dread of fever which is often observed to hang on their minds, and which tends not a little to aggravate symptoms, if not perhaps to induce disease.

The practice of bleeding in the fever in question, has now but one abetter among the Medical Gentlemen of Kingston; and he, I suspect, in recommending it, is actuated more by doctorial vanity than by a conviction of its utility. For, after having boasted that he has practised here upwards of thirty years, he could not stoop to acknowledge himself fallible, or even submit to have his opinion called in question.

In the summer of the year 1794, this fever raged with so great malignancy, and the remedies hitherto employed were productive of so little good, that practitioners were eager to try any thing, however doubtful the success. Such was the horror excited by the dreadful mortality which prevailed, that no sooner was bleeding spoken of than every one had recourse to it; even those whose opinions were decidedly against it as a remedy in idiopathic fever. The situation I then held in Kingston, obliged me to be very frequently an operator in such cases. In some the bleeding produced a slight alleviation of the distressing head-ach and general pains, but of short duration, these symptoms resuming soon their former severity. The relief here observed, seemed altogether to arise from a strong disposition which there was in most cases to syncope, even though bled in the recumbent posture. It would be well if the advocates for bleeding did but observe attentively the ultimate as well as the more immediate effects of taking away blood; for though this, by inducing syncope, and succeeding languor, or an approach to syncope, may for the moment abate those symptoms connected with the inordinate action of the heart and arteries; yet the disposition which it gives to debility, sufficiently great without such aid, ought to compel every practitioner to weigh well, whether the temporary mitigation of some symptoms compensates the subsequent bad effects. It is somewhat remarkable, that at the time I speak of, the greatest harm appeared to arise from the use of the lancet in the cases which seemed most to require it. In proportion as the patient was full, robust and vigorous, and the attack of fever severe, so in general was the rapidity and degree of the debility which succeeded the abstraction of blood. The most astonishing case of this kind which occured, was that of a seaman, the particulars of which you may recollect having been laid before the medical meetings then held in this place. He was a remarkably markably stout young man, nineteen years of age, in whom the symptoms of head-ach, flushed face, redness of the eyes, and general uneasiness were very severe. It so happened, that other patients were visited on board the same ship the morning he was taken ill, by which the earliest opportunity was given* for bleeding. At the time the things necessary for performing the operation were getting ready, he was still walking about, but was desired to sit down on a mattrass placed on the cabin floor as his bed. A vein was opened in the arm while sitting; but no sooner had he lost three or four ounces of blood than supervening syncope compelled him to lie down. In this posture, when about eight ounces more were taken away, he became so extremely languid as to make it advisable to tie up the arm. He never again got up, for, from that moment he continued feeble, and debility advanced progressively in spite of every thing given to obviate it, till the third day, when he died. Though it is possible this case might, under any treatment, have terminated in death, is there not reason to think that things were brought much sooner to a fatal crisis by the loss of blood? It seemed as if the powers of life had been so exhausted by it, as to be incapable of being again roused into action. The lancet was employed without evident mischief in the most favourable cases only; those in which the symptoms were so mild, that the patient must have done well under any treatment, or if left to himself. The practice could not have a fairer trial, nor a better opportunity of being brought into repute, had it been found beneficial, than at the time here alluded to: the general result convinced all the practitioners then resident in Kingston, whose opinions were worth taking, that bleeding is a remedy which in no case of the fever of new-comers is productive of good; but in most cases, of much evil.

I am, &c.

*Kingston, Sept.* 20, 1802. CHARLES MAC LARTY.

---

*Extract of a Letter from* Dr. Burke.

Dear Sir,

Subjoined I send you the names of those persons with whom I have employed mercury as a prophylactic against the malignant pestilential fever of this country. Some were seized with it at an earlier, others at a later period, after the use of mercury, and all recovered. But I have constantly remarked, that those who laboured under ptyalism, ptyalism, at the first attack of fever, had much milder symptoms, and recovered more speedily. I have treated many more according to this plan with equal success, but cannot, at present, bring their names to my recollection.

I am, Dear Sir, &c.

*Kingston, Oct.* 11, 1802. WM. BURKE.

*To Doctor* JOHN HARRIS.

Names of those treated according to the prophylactic plan.

| | |
|---|---|
| J. P. Malony, Esq. | P. Aylward |
| James Dewdney | Thomas Shea |
| Wm. Coppinger | James Shea |
| Ludovick Morisey | William Gow |
| James Tyrell | Rev. Mr. Supple |
| Edward Tyrell | Robert Blair. |

